# P-2300. Invasive Pulmonary Aspergillosis-Mimicking *Pseudomonas aeruginosa* Pneumonia in Immunocompromised Patients

**DOI:** 10.1093/ofid/ofae631.2453

**Published:** 2025-01-29

**Authors:** Choseok Yoon, Euijin Chang, Seongman Bae, Jiwon Jung, Min Jae Kim, Yong Pil Chong, Sang-Oh Lee, Sang-Ho Choi, Yang Soo Kim, Sung-Han Kim

**Affiliations:** Hanyang University Seoul Hosptial, Seoul, Seoul-t'ukpyolsi, Republic of Korea; Asan medical center/Department of Infectious disease, Seoul, Seoul-t'ukpyolsi, Republic of Korea; Asan medical center/Department of Infectious disease, Seoul, Seoul-t'ukpyolsi, Republic of Korea; Asan Medical Center, Seoul, Seoul-t'ukpyolsi, Republic of Korea; Asan Medical Center, Seoul, Seoul-t'ukpyolsi, Republic of Korea; Asan Medical Center, Seoul, Seoul-t'ukpyolsi, Republic of Korea; Asan Medical Center, Seoul, Seoul-t'ukpyolsi, Republic of Korea; Asan Medical Center, Seoul, Seoul-t'ukpyolsi, Republic of Korea; Asan Medical Center, Seoul, Seoul-t'ukpyolsi, Republic of Korea; Asan medical center, Seoul, Seoul-t'ukpyolsi, Republic of Korea

## Abstract

**Background:**

*Pseudomonas aeruginosa* (PA) pneumonia is occasionally misdiagnosed as invasive pulmonary aspergillosis (IPA) in immunocompromised patients, especially transplant recipients or those with hematologic malignancy who have risk factors for IPA. However, there are limited data on this area. We thus investigated the clinical features and CT findings of PA pneumonia in immunocompromised patients.

**Figure:**
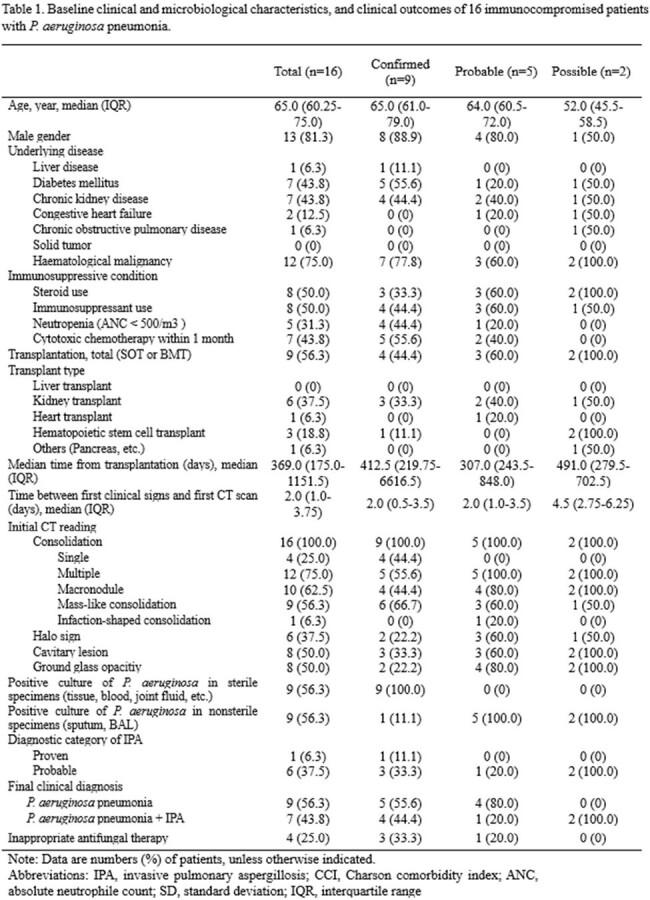

**Methods:**

All immunocompromised patients who were diagnosed as PA pneumonia was retrospectively reviewed in a tertiary hospital between 2017 and 2023. PA pneumonia was classified as follows; “confirmed”: positive culture from sterile tissues (blood, tissue, etc.) with radiologic compatible findings with pneumonia, “probable”: i) abnormalities on chest X-ray, ii) culture growth from non-sterile sources such as sputum or bronchoalveolar lavage fluid, iii) improvement with the use of anti-pseudomonal antibiotics without the use of antifungal agents, and “possible”: cases that do not meet the above criteria but cannot rule out PA pneumonia.

**Figure:**
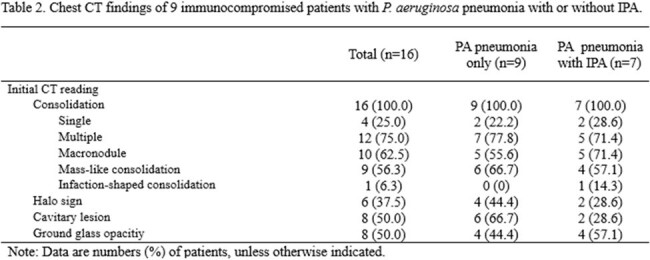

**Results:**

Of 16 immunocompromised with PA pneumonia, 13 (81%) were male, and 9 (56%) were transplant recipients. Of these 16 patients, 9 (56%) were classified as “confirmed”, 5 (31%) “probable”, and the remaining 2 (13%) “possible”. 7 (44%) patients with PA pneumonia had concurrent IPA, with 1 proven IPA and 6 probable IPA. The remaining 9 patients with PA pneumonia alone revealed macronodules (n=5), mass-like consolidation (n=6), cavitary lesions (n=6), and halo sign (n=4) in initial CT findings. Of the 16 patients with PA pneumonia, 4 (25%) received inappropriate antifungal therapy.

**Conclusion:**

About half of immunocompromised patients with PA pneumonia had concurrent IPA, but about one quarter of immunocompromised patients with PA pneumonia received inappropriate antifungal therapy.

**Disclosures:**

All Authors: No reported disclosures

